# The Synthetic Melanocortin (CKPV)_2_ Exerts Anti-Fungal and Anti-Inflammatory Effects against Candida albicans Vaginitis via Inducing Macrophage M_2_ Polarization

**DOI:** 10.1371/journal.pone.0056004

**Published:** 2013-02-14

**Authors:** Hai-xia Ji, Yu-lian Zou, Jing-jing Duan, Zhi-rong Jia, Xian-jing Li, Zhuo Wang, Li Li, Yong-wen Li, Gen-yan Liu, Ming-Qing Tong, Xiao-yi Li, Guo-hui Zhang, Xiang-rong Dai, Ling He, Zhi-yu Li, Cong Cao, Yong Yang

**Affiliations:** 1 State Key Laboratory of Natural Medicines, Academic Institute of Pharmaceutical Science, China Pharmaceutical University, Nanjing, Jiangsu, People’s Republic of China; 2 Department of Pharmacology, Department of Physiology, Guilin Medical University, Guilin, Guangxi, People’s Republic of China; 3 Department of Laboratory Medicine, the First Affiliated Hospital of Nanjing Medical University, Nanjing, Jiangsu, People’s Republic of China; 4 Neuroscience Institute, Soochow University, Soochow, Jiangsu, People’s Republic of China; 5 Hefei Zhaoke Pharmaceutical, Hefei, People’s Republic of China; University of California Merced, United States of America

## Abstract

In this study, we examined anti-fungal and anti-inflammatory effects of the synthetic melanocortin peptide (Ac-Cys-Lys-Pro-Val-NH_2_)_2_ or (CKPV)_2_ against Candida albicans vaginitis. Our *in vitro* results showed that (CKPV)_2_ dose-dependently inhibited Candida albicans colonies formation. In a rat Candida albicans vaginitis model, (CKPV)_2_ significantly inhibited vaginal Candida albicans survival and macrophages sub-epithelial mucosa infiltration. For mechanisms study, we observed that (CKPV)_2_ inhibited macrophages phagocytosis of Candida albicans. Meanwhile, (CKPV)_2_ administration inhibited macrophage pro-inflammatory cytokines (TNF-α, IL-1β and IL-6) release, while increasing the arginase activity and anti-inflammatory cytokine IL-10 production, suggesting macrophages M1 to M2 polarization. Cyclic AMP (cAMP) production was also induced by (CKPV)_2_ administration in macrophages. These above effects on macrophages by (CKPV)_2_ were almost reversed by melanocortin receptor-1(MC1R) siRNA knockdown, indicating the requirement of MC1R in the process. Altogether, our results suggest that (CKPV)_2_ exerted anti-fungal and anti-inflammatory activities against Candida albicans vaginitis probably through inducing macrophages M1 to M2 polarization and MC1R activation.

## Introduction

The common commensal Fungus Candida albicans cause systemic or mucocutaneous infections in abnormal immunity environments [1]. Vulvovaginal candidiasis (VVC) is a frequent mucosal infection caused by Candida species, which affects a number of women in child-bearing years. 75% of all women will experience at least one acute VVC infection during their lifetime, and 40%–50% of them could be infected twice or more. Several known predisposing factors including antibiotic, oral contraceptive usage, hormone replacement therapy, pregnancy, uncontrolled diabetes mellitus, and possible HIV infection increase the susceptibility of Candida albicans [2], [3]. A small population of women (∼5%–10%) has recurrent vulvovaginal candidiasis (RVVC) [4].

80%–90% VVC patients are treated with imidazole drugs, which can relieve symptoms and prevent inflammations. However, these drugs usually have side effects including itching, burning, local allergic reactions and other possible off-target toxicities. Other medicines like Ketoconazole could potentially cause systemic toxicity to the patients, and therapeutic dose of triazole is not able to kill all Candida species [5]–[7].

Alpha-melanocyte-stimulating hormone (α-MSH) is a neuroendocrine-immune regulatory peptide. It is composed of 13 peptides (N-Aeetyl-Ser-Tyr-Ser-Met-Glu-His-Phe- Arg-Trp-Gly-Lys-Pro-val-NH_2_). Recent literatures have studied its anti-microbial [8]–[10] and anti-inflammatory effects [11]–[13]. There are at least five α-MSH receptors, namely melanocortin receptor1–5 (MC1–5R). When activated, these G protein-coupled receptors (GPCR) stimulate adenylate cyclase (AC), and induce intracellular cyclic AMP (cAMP) production. α-MSH is known to bind to all melanocortin receptors with strong affinities except MC2R [14].

α-MSH shows significant anti-microbial and anti-inflammatory effects. In macrophages, α-MSH activates MC1R and inhibits lipopolysaccharide (LPS)-induced nuclear factor κB (NF-κB) activation [15]. Similarly, α-MSH_11–13_(KPV), the C-terminal tripeptide of α-MSH, also has a wide range of anti-microbial and anti-inflammatory activities. However, KPV could inhibit inflammation with no cAMP accumulation, suggesting that its anti-inflammatory effects may not be solely dependent on MCRs [16].

(CKPV)_2_, (Ac-Cys-Lys-Pro-Val-NH2)_2_, similar to α-MSH in structure [17], is synthesized by inserting a Cys-Cys linker between the two units of KPV. Catania et al., first showed its excellent anti-Candidacidal effects [18], following studies focused on its anti-inflammatory effects. In a mouse model peritonitis-induced by LPS, (CKPV)_2_ administration markedly decreased circulating TNF-α and NO_2_^−^ in plasma and peritoneal cavity [19]. *In vivo* and *in vitro* experiments demonstrated that (CKPV)_2_ could prevent human neutrophils migration, reactive oxygen intermediate (ROI) production, pro-inflammatory cytokines (interleukin 1β or IL-1β, tumor necrosis factor or TNF-α) secretion and adhesion molecules (ICAM-1) expression [10], [20], [21]. The fact that cAMP inhibitor abolished (CKPV)_2_’s effects on chemo-taxis and respiratory burst [20] suggests that the anti-inflammatory activity of (CKPV)_2_ may be dependent on MCRs, as similar to α-MSH.

Macrophages serve as essential sentinels in innate immunity and effectors in the transition to adaptive immunity. Macrophages participate in immune regulation and tissue repair depending on the environmental status. They present various activated types ranging from classically activated M1 macrophages to alternatively activated M2 macrophages [22]. M1 macrophages are associated with the expression of inflammatory factors, such as interleukin 1β (IL-1β), IL-12, inducible nitric oxide synthase (iNOS), and have considerable roles in counteracting microbial product [22]. On the other hand, M2 macrophages secret anti-inflammatory mediators in response to wound healing, host defense against inflammation [22]. In our study, we found that (CKPV)_2_ not only played a Candidacidal role in rat Candida albicans vaginitis, but also induced anti-inflammatory effects by inducing macrophages M2 polarization, activation of MC1R appeared is involved in the process.

## Materials and Methods

### Reagents

(CKPV)_2_ and α-MSH were synthesized and provided by Hefei Zhaoke Pharmaceutical Co. Ltd.

### Primary Culture of Mouse Peritoneal Macrophages

Primary culture of mouse peritoneal macrophages was similar as previously described [23], the generation of macrophages was induced by intra-peritoneal injection 3 mL of 3% thioglycolate (Sigma Aldrich Fluka, USA) dissolved in phosphate-buffered saline (PBS). After three days, the mice were sacrificed, immersed with 75% ethanol for 2–3 minutes, immobilized on the autopsy table. The abdominal skin was exposed and 5 ml of PBS was injected into the peritoneal cavity and gently knead for 1–2 min, abdominal membrane was lift by forceps and leaned to one side to focus the fluid in the abdomen. The primary mouse peritoneal macrophages suspension were collected into tubes and centrifuged at 1000 rpm at 4°C for 5 minutes, the supernatant was then removed. The cells were further washed and suspended (10^6^/ml) with DMEM complete culture medium which contain 10% fetal calf serum (FCS) with penicillin/streptomycin. They were incubated at 37°C for 2 h and the adherent cells were macrophages.

### Candida Albicans Colony Formation

Candida albicans (from Jiangsu Province Hospital, Nanjing, China) were cultured in 5 ml liquid Luria-Bertani (LB) medium and amplified for 12–16 hours, washed twice with distilled water, suspended in liquid LB medium to a final concentration of 10^7^/ml and incubated at 30°C for 2 hours in the presence of different concentrations of (CKPV)_2_ or vehicle control. To determine the inhibitory effect of (CKPV)_2_, the Candida albicans in the absence and presence of (CKPV)_2_ were inoculated to Sabouraud medium and cultured at 30°C for 48 h. The amounts of colonies and the inhibition rate were calculated as previously reported [3], [8], [9].

### Rat Candida Albicans Vaginitis

Sixty female SD rats, weighing 180–220 g, were maintained in pseudo-estrus by intragastric administration of estradiol valerate (0.8 mg/kg) per day. Four days after first administration, the rats were inoculated intravaginally with 4×10^7^ yeast cells (0.1 ml) according to established procedures [24]–[26]. To assess the anti-fungal activity of (CKPV)_2_, the rats were treated with blank matrix gel (0.2 ml/rat), miconazole gel (0.5 mg/kg ), α-MSH gel (1.7 mg/kg) or (CKPV)_2 _gel (2, 1 and 0.5 mg/kg) respectively per day. Vaginal fluid was collected from each animal at 0, 3, 11, 18 days after administration to calculate the colony forming units (CFUs). At the end of the experiments, all the rats were sacrificed and their vaginal tissues were dissociated for immunohistochemistry examination. This study was carried out in strict accordance with the recommendations in the Guide for the Care and Use of Laboratory Animals of the NSFC. The protocol was approved by the Committee on the Ethics of Animal Experiments of the Nanjing Pharmaceutical University. All surgery was performed under proper anesthesia, and all efforts were made to minimize suffering.

### Immunohistochemistry

Formalin-fixed paraffin-embedded vaginal tissue samples were used for analysis of macrophages infiltration as previously described [27]. Anti-CD68 (ab31630, dilution 1∶200;Abcam, Cambridge) Anti-CD163 (ab119996, dilution 1∶250, Abcam, Cambridge) were applied to label CD68 and CD163 as makers for M1 and M2 macrophages separately. The immune-fluorescence images were captured on a LEICA DMI3000 B confocal microscope, using 10× and 40× objective.

### Candida Albicans Phagocytosis by Activated Peritoneal Macrophages

The macrophages were seeded into 24-well plates (2×10^5^/well) for 4 h and incubated with PBS, LPS (5 ng/ml)/IFN-γ(10 ng/ml), α-MSH (10^−6 ^M) or indicated concentration of (CKPV)_2_ respectively for 24 h. Heat-inactivated Candida albicans were washed twice with PBS, centrifuged at 1000 rpm for 5 min and stained with Giemsa dye reagent (Jiancheng Technology Co. Ltd, Nanjing, China). The suspension containing 2×10^7^ Candida albicans was added to each well containing macrophages. The plates then were carefully incubated at 37°C for 1 h. After extensively washes, the number of cells engulfed Giemsa dye stained- Candida albicans was recorded under the microscope [28].

### MC1R Exogenously Expression in COS-7 Cells

Mouse MC1R cDNA extracted from B16-F10 cells (ATCC, Maryland, USA) was sub-cloned into Amp+ enhancer and promoter reporter vector PRL14.4 to yield PRL14.4-MC1R. Then the MC1R gene was transferred into Phage transfer vector pGEM-T4. The recombinant plasmid was transfected into COS-7 cells as previously described [29], [30].

### MC1R RNA Interference (RNAi)

The chemically synthesized MC1R siRNA (small interfering RNA) duplexes (Tab. 1) against mouse MC1R (s1, s2 and s3) were purchased from Ambion (GenePharm Co. Ltd. Shanghai, China). The primary cultured macrophages were plated in 24-well plates (2.5×10^5^/well). After 4 h, siRNA was transfected into cells with Lipofectamine 2000 according to the manufacturer^’^s protocol (Invitrogen). 48 hours after transfection, the mRNA level of MC1R in transfected cells was detected to test RNAi efficiency [15], [31].

**Table 1:** Mouse MC1R siRNA duplex sequences.

Name	Reference No	Sense sequence (5′-3′)	Antisense sequence (5′-3′)
GAPDH	–	CACUCAAGAUUGUCAGCAATT	UUGCUGACAAUCUUGAGUGAG
MC1R-siRNA(S1)	Mc1r-mus-1243	GCUGCAUCUUCAAGAACUUTT	AAGUUCUUGAAGAUGCAGCTT
MC1R-siRNA(S2)	Mc1r-mus-949	GCACCCUCUUUAUCACCUATT	UAGGUGAUAAAGAGGGUGCTT
MC1R-siRNA(S3)	Mc1r-mus-694	GUGCUGGAGACUACUAUCATT	UGAUAGUAGUCUCCAGCACTT
Negative control(NC)	–	UUCUCCGAACGUGUCACGUTT	ACGUGACACGUUCGGAGAATT

### cAMP Assay

The primary cultured macrophages were seeded in 24 well plates in DMEM supplemented with 10% FCS and incubated at 37°C for 2 hours for adhesion. Thirty min after indicated treatment/s [32], [33], the cells were lysed with 0.1 M HCl for 20 min. The cAMP levels were determined with mouse cyclic adenosine monophosphate (cAMP) ELISA Kit (Abcam, UK) based on the manufacturer^’^s protocol.

### TNF-α Cytotoxicity Assay

The supernatant (100 µl/well) of the macrophages after indicated treatment/s was added to L929 cells for 20 h [34], [35]. MTT (3-(4,5-dimethylthazol-2-yl)-2,5-diphenyltetrazolinum bromide) salt (0.25 mg/ml) was added to each well. Afterwards, L929 cells were further incubated in CO_2_ incubator for 4 hours at 37°C, 100 µl of DMSO was then added to dissolve formazan crystals and the absorbance of each well was observed by a plate reader at a test wavelength of 570 nm. The OD value was normalized to untreated vehicle control group.

### Arginase Activity Assay

Triton X-100 (0.1%, 100 µl) was added to the macrophages with indicated treatment/s. After 30 min incubation, tris-HCl and MnCl_2_ mixture (100 µl) were added to cells at 56°C for 10 min. The plates were incubated with L-arginine (100 µl) at 37°C for 30 min, and then H_2_SO_4_/H_3_PO_4_/H_2_O mixture (800 µl) was added and heated with α-isopropylidene nitrobenzene acetone (50 µl) at 95°C for 30 min. The complex in each well was diluted 20 times with PBS to detect the optical density (OD) values at 540 nm with a UV spectrophotometer [36], [37].

### IL-1β, IL-6 and IL-10 ELISA assay

The macrophages supernatant was collected 24 hours after indicated treatment/s. TNF-α,IL-1β, IL-6 and IL-10 levels were determined via double antibodies sandwich ABC-ELISA kits from R&D Systems (Minneapolis, USA) according the manufacturer procedures.

### Statistical Analysis

Individual culture dishes or wells were analyzed separately (no pooling of samples was used). In each experiment a minimum of six wells/dishes of each treatment was used. Each experiment was repeated a minimum of three times. In each experiment, the mean value of the repetitions was calculated and this value was used in the statistical analysis. Data are presented as mean ± SEM. The differences were determined by one-way anova in appropriate experiments followed by Newman–Keuls *post hoc* test. A probability value of ***p***<0.05 was taken to be statistically significant.

## Results

### (CKPV)_2_ inhibits Candida Albicans SA-40 Colonies Formation

**Figure 1:**
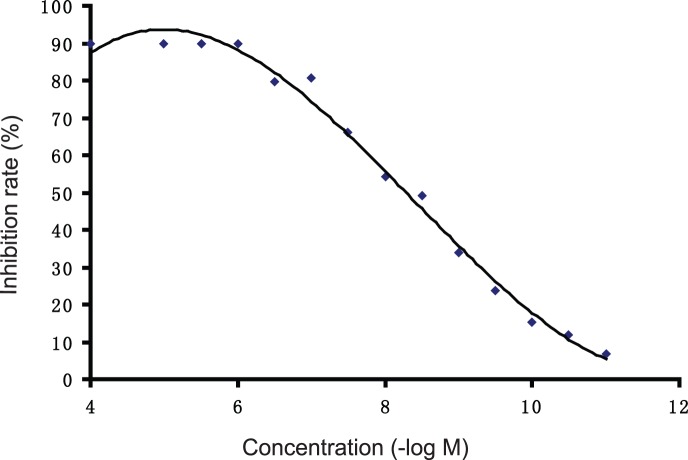
(CKPV)_2_’s inhibits Candida albicans SA-40 colonies formation. The inhibitory rate of different concentrations of (CKPV)_2_ against Candida albicans SA-40 in vitro. Candida albicans were incubated with PBS (vehicle control) or indicated concentration of (CKPV)_2_ (10^−11 ^M, 3×10^−10 ^M, 10^−10 ^M, 3×10^−9 ^M, 10^−9 ^M, 3×10^−8 ^M, 10^−8 ^M, 3×10^−7 ^M, 10^−7 ^M, 3×10^−6 ^M, 10^−6 ^M, 3×10^−5 ^M, 10^−5 ^M and 10^−4 ^M) at 30°C for 2 h, afterwards, Candida albicans were transferred to Sabouraud medium and cultured at 30°C for 48 h. The inhibitory ratio(%) = the CFUs of Candida albicans treated with(CKPV)_2_/the CFUs of Candida albicans treated with PBS ×100. Experiments in this figure were repeated at least three times and similar results were obtained.

To detect whether (CKPV)_2_ has the capacity to inhibit the Candida albicans directly, we first examined the anti-fungal effects of (CKPV)_2_ *in vitro*. Results in Fig. 1 showed that (CKPV)_2_ dose-dependently inhibited Candida albicans colonies formation. The fungistatic rate was up to 50% and 90% after 3×10^−8^ M and 10^−6^ M (CKPV)_2_ exposure respectively (Fig. 1). These results suggest that (CKPV)_2_ could directly inhibit Candida albicans SA-40.

### (CKPV)_2_ Inhibits Candida Albicans in a Rat Vaginitis Model

**Figure 2:**
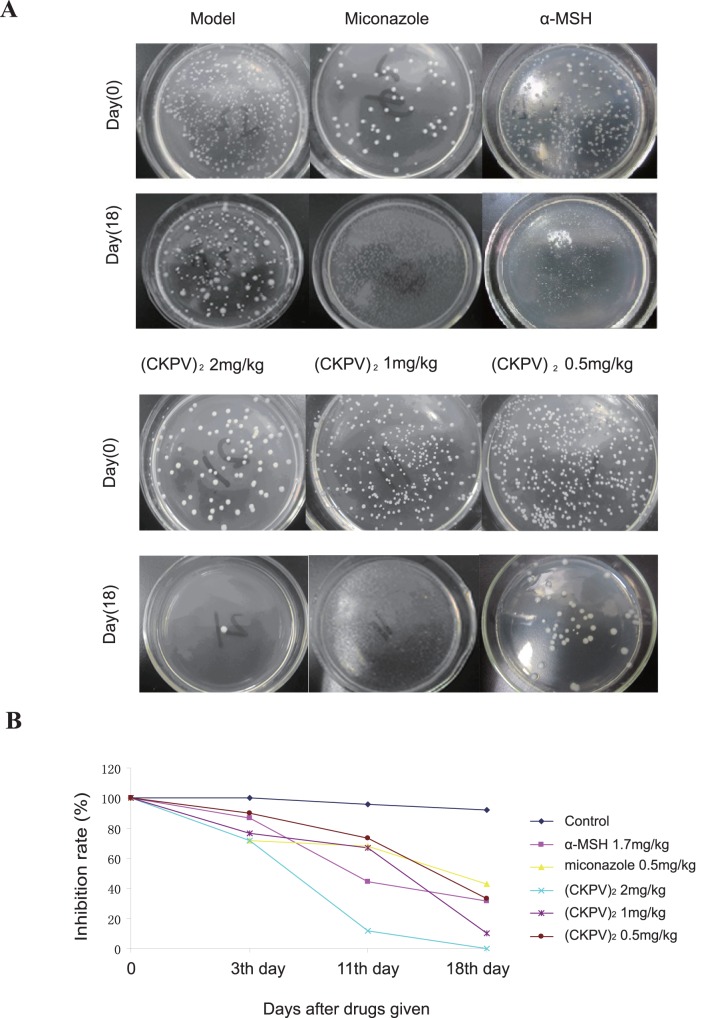
(CKPV)_2_ inhibits Candida albicans in a rat vaginitis model. (A) The CFUs of Candida albicans at day 0 and day 18 after indicated treatment. (B) The inhibitory ratio of (CKPV)_2_ to Candida albicans survival. The survival ratio (%) was calculated by the number of surviving colonies after indicated drug treatment divided by the number of colonies before treatment. Experiments in this figure were repeated three times and similar results were obtained.

A rat Candida albicans vaginitis model was applied to study the anti-fungal activities of (CKPV)_2_ *in vitro*. Results showed that (CKPV)_2_ administration exerted significant anti-Candida albicans vaginitis effects. (CKPV)_2_ at 2 mg/kg showed the strongest inhibition against vaginal Candida albicans, as the survival of Candida albicans dropped to 12.0% at the 11th day of the treatment, while the survival rate of miconazole (0. 5 mg/kg)-treated group was 44.7%. At the 18th day of treatment, the survival rate of vaginal Candida albicans of (CKPV)_2_ (2 mg/kg)-treated group was close to 0% (Fig. 2). The results suggested that (CKPV)_2_ were more effective against Candida albicans vaginitis than α-MSH or miconazole (Tab. 2, Fig. 2).

**Table 2:** The effects of (CKPV) 2 on Candida albicans in rat vaginitis (n = 6).

Group	Doses(mg/kg)	CFUs, Mean degree and Survival ratio(%)of C.albicans on day(X)											
		0			3			11			18		
		CFUs	Mean degree	Survival ratio(%)	CFUs	Mean degree	Survival ratio(%)	CFUs	Mean degree	Survival ratio(%)	CFUs	Mean degree	Survivalratio(%)
Control	-	>200	2.5	100.0	>200	2.5	100.0	>200	2.4	96.0	>200	2.3	92.0
Miconazole	0.5	>200	3.8	100.0	>200	3.3	86.8	150	1.7	44.7	80	1.2	31.6
α-MSH	1.7	100	2.5	100.0	79	1.8	72.0	77	1.7	68.0	43	1.2	42.9
(CKPV) _2_	2	>200	2.5	100.0	39	1.8	72.0	10	0.3	12.0	1	0.0	0.0
(CKPV)_2_	1	386	3.0	100.0	123	2.3	76.7	55	2.0	66.7	8	0.3	10.0
(CKPV) _2_	0.5	262	3.0	100.0	105	2.7	90.0	283	2.2	73.3	24	1.0	33.3

At day 0, 3, 11 and 18 after indicated treatment (per day), vaginal was lavaged with 100 µl PBS, afterwards, the lavage was transferred to Sabouraud medium after dilution of 1∶1000 and cultured at 30°C for 48 h. Colonies forming units (CFUs) were recorded and classified according to the following standard: the number of colonies >1000, degree 4; 100–1000, degree 3; 10–100, degree 2; 5–10, degree1; <5, degree 0.

### In a Rat Vaginitis Model, (CKPV)_2_ Promotes Infiltrated Macrophage M2 Polarization

**Figure 3:**
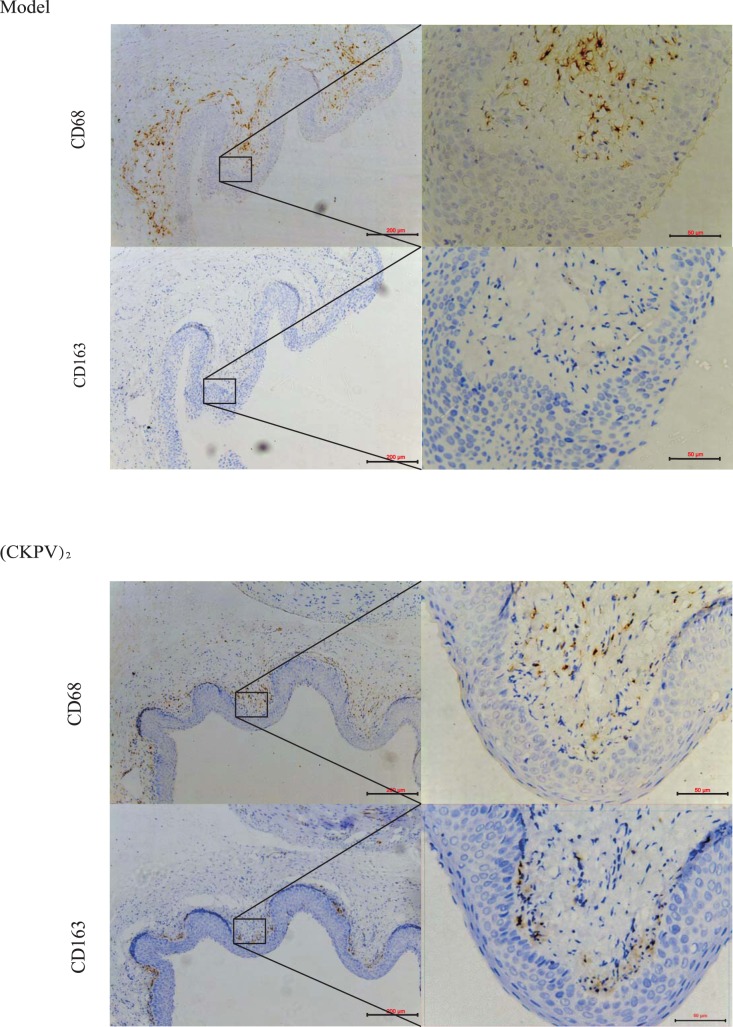
In a rat vaginitis model, (CKPV)2 promotes infiltrated macrophage M2 polarization. CD68 and CD163 staining in the vehicle control (upper panel) and (CKPV)**_2_**-treated (lower panel) group. Bar = 50 µm (Left); Bar = 200 µm (Right). Experiments in this figure were repeated three times and similar results were obtained.

Studies have been focusing on the underlying mechanism by which host responses to Candida albicans infections. Infiltration of inflammatory cells, mainly polymorphonuclear leukocytes and macrophages, and/or some lymphocytes has been proposed. These infiltrated inflammatory cells response to microorganism invasion via secreting multiple cytokines to trigger inflammation. To determine the anti-inflammatory effect of (CKPV)_2,_ we again employed rat models of experimental vaginitis. The vaginal fungal burden (in CFU) was measured as the indicator of the level of infections, the mucosa infiltrate immune cells after Candida albicans infection were examined via immunohistochemistry [38], [39]. Our results showed that the infiltrated immune cells in model group were mainly M1 macrophages (CD 68 positive) with few M2 (CD 163 positive) macrophages. On the other hand, in the (CKPV)_2_-treated group, M2 macrophages (CD 163 positive) were the main infiltrated cells (Fig. 3), indicating that (CKPV)_2_’s anti-inflammatory effects may through inducing macrophages M1to M2 polarization.

### (CKPV)_2_ Inhibits Macrophages Phagocytosis of Candida Albicans

**Figure 4:**
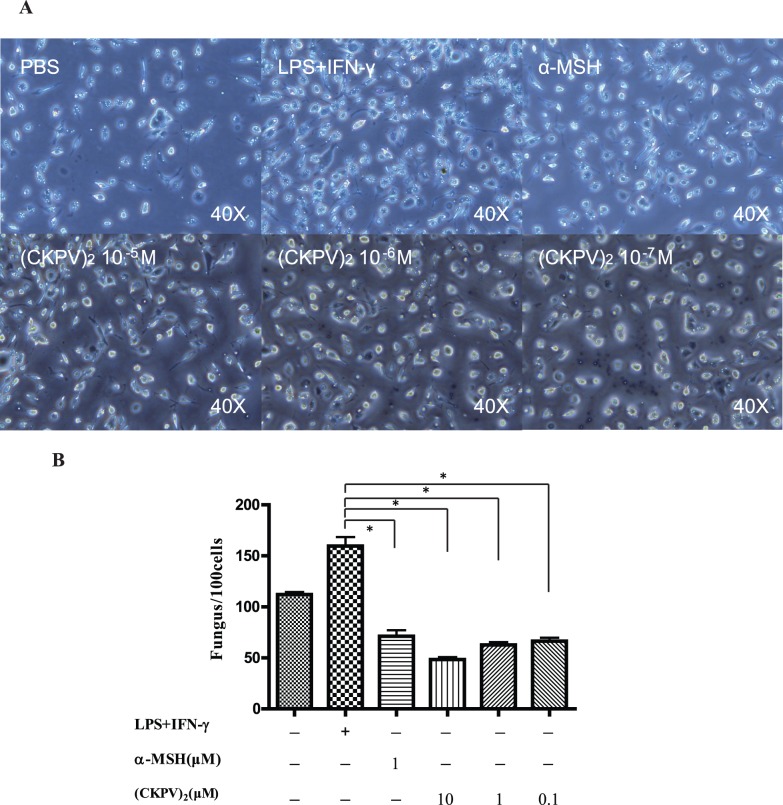
(CKPV)_2_ inhibits macrophages phagocytosis of Candida albicans. (A) The macrophage cells (wide arrow) were polygonal shape stretching and Candida albicans (tiny arrow) were dyed bright blue by Wright-Ji Musa in inverted microscope. (Magnification: 40). The number of cells and engulfed microorganism were recorded in 10 random views under the microscope. (B) The phagocytosis index (number of engulfed fungus/100 cells)of macrophages with indicated treatment. *p<0.01 (ANOVA). Experiments in this figure were repeated three times and similar results were obtained.

To study the underlying mechanism of (CKPV)_2_-induced anti-fungal and anti-inflammatory effects against Candida albicans, we examined (CKPV)_2_’s effects on primary cultured macrophages. We found that both α-MSH and (CKPV)_2_ significantly inhibited Candida albicans phagocytosis by interferon γ (IFN-γ)/LPS-activated macrophages (Fig. 4), suggesting that (CKPV)_2_ directly inhibits phagocytosis ability of primary cultured macrophages.

### (CKPV)_2_ Promotes cAMP Production via MC1R

**Figure 5:**
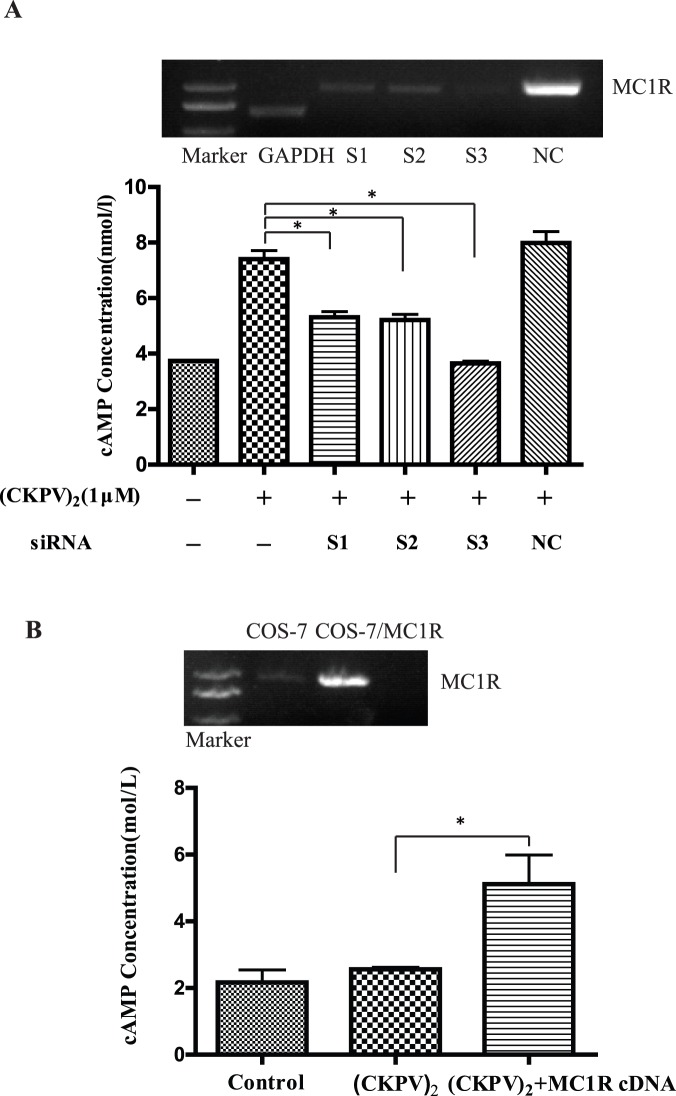
(CKPV)_2_ promotes cAMP production via MC1R. (A) Upper panel: the effects of MC1R siRNA (S1, S2 and S3, see sequence on Tab. 1) on MC1R mRNA level in primary cultured macrophages. Lower panel: MC1R siRNA knockdown almost blocked (CKPV)_2_-induced cAMP production in macrophages. (B) Upper panel, RT-PCR results confirms (CKPV)_2_ mRNA expression in MC1R cDNA-transfected COS-7 cells (COS-7/MC1R) or nonsense-cDNA-transfected control cells (COS-7). Lower panel: (CKPV)_2_-induced cAMP production in negative control-cDNA-transfected (NC) or MC1R cDNA-transfected COS-7 cells. *p<0.01 (ANOVA). Experiments in this figure were repeated three times and similar results were obtained.

Studies have shown that melanocortin peptides cause cAMP production via activating melanocortin receptor-1(MC1R) in macrophages. We then examined whether (CKPV)_2_ had the similar effects. Results showed that the cAMP level was significantly augmented in primary cultured macrophages after (CKPV)_2_ treatment. To demonstrate the role of MC1R in this process, we applied target siRNAs to effectively knockdown MC1R in macrophages (Fig. 5A). Results showed that knockdown of MC1R by target siRNAs (S1, S2 and S3) (see RNAi sequence in Tab. 1) significantly reduced (CKPV)_2_-induced cAMP production in macrophages (Fig. 5A). However, no cAMP was induced in MC1R-null COS-7 cells after (CKPV)_2_ exposure. On the other hand, cAMP level was increased in COS-7 cells exogenously expressing MC1R. (Fig. 5B). These results together suggest that (CKPV)_2_ promotes cAMP production via MC1R.

### (CKPV)_2_ Induces Macrophage M1 to M2 Polarization

**Figure 6:**
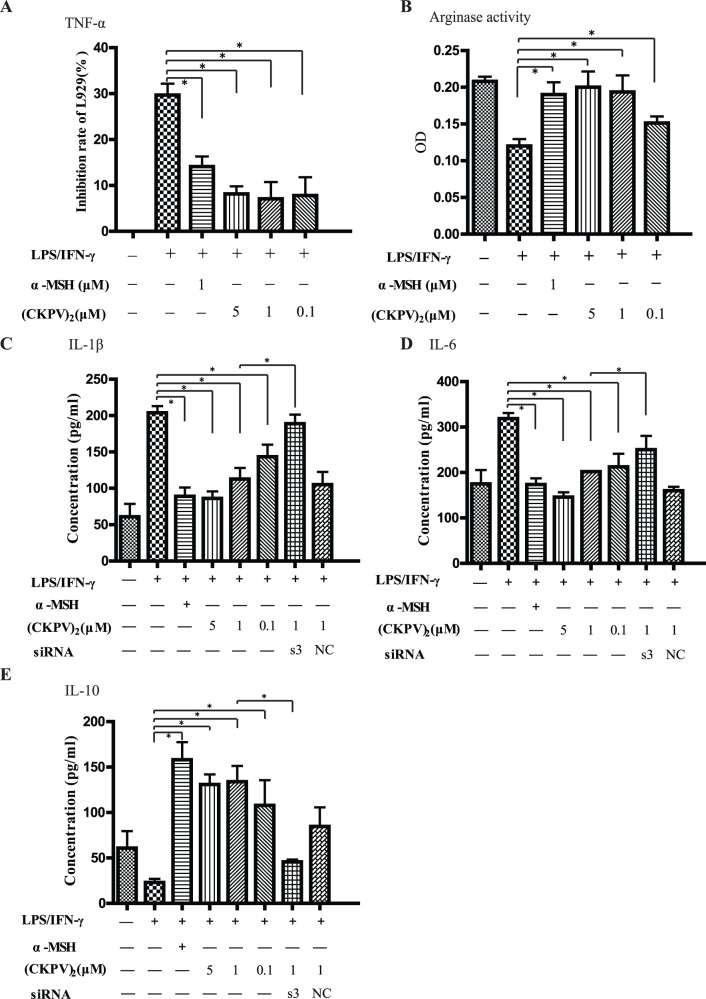
(CKPV)_2_ induces macrophage M1 to M2 polarization. Cytokine release profile (IL-1β, IL-6 and IL-10) and arginase activity after indicated treatment/s in primary cultured macrophages transfected with or without MC1R RNAi. LPS:5 ng/ml, IFN-γ:10 ng/ml, α-MSH: 10 µM, and (CKPV)_2_ (0.1, 1 and 5 µM) (B–E). The supernatant of the above macrophages were collected and added to L929 cells, after 20 hours, cell viability was measured by MTT assay, the inhibitory rate was calculated by 100%-cell viability OD of treatment group/cell viability OD of untreated control group (A). *p<0.01 (ANOVA). Experiments in this figure were repeated three times and similar results were obtained.

Macrophage M1 to M2 polarization involves a reduction in expression of pro-inflammatory cytokines, together with an increase of IL-10 secretion and arginase activity [40]. Here, we observed that LPS/IFN-γ-induced TNF-α production was almost blocked by co-administration with (CKPV)_2_ in primary cultured macrophages, as the cytotoxicity of (CKPV)_2_-treated macrophages supernatant to L929 cells decreased significantly (Fig. 6A). The secretion of inflammatory cytokines including IL-1β and IL-6 was also inhibited after (CKPV)_2_ administration (Fig. 6C and D). Importantly, macrophage IL-10 production and arginase activity, the indicators of M2 polarization, were significantly increased by (CKPV)_2_ (Fig. 6B and E). Notably, (CKPV)_2_’s effects on cytokines production were almost blocked by MC1R siRNA knockdown, suggesting that MC1R was required for (CKPV)_2_ effects on macrophages (Fig. 6C–E). It should be noted that α-MSH had similar effects on macrophages as (CKPV)_2_ (Fig. 6). Based on these data, we summarized that (CKPV)_2_ inhibited pro-inflammatory cytokines (TNF-α, IL-1β and IL-6) production while increasing arginase activity and the secretion of IL-10 to favor a macrophage M1 to M2 polarization.

### Anti-acute Inflammatory Effects of (CKPV)_2_

Above results confirmed the potential anti-fungal effects of CKPV)_2_, we also tested (CKPV)_2_’s potential role in other inflammation models including mouse ear edema, rat paw edema and rat foot itching (see protocol intext S1). Results were included in Fig. S1. The mice right ears became evident swelling and flare after xylene administration. Dexamethasone, α-MSH and (CKPV)_2_ significantly suppressed these inflammation reactions (Fig. S1-A). Subcutaneous injection of albumen into right paw caused immediate and persistent edema with the peak at 0.5 h–1 h. Dexamethasone, α-MSH and (CKPV)_2_ (high and middle doses) relieved paw edema (Fig. S1-B). Phosphate-induced itching was also blocked by dexamethasone, α-MSH and (CKPV)_2_ (high and middle doses) (Fig. S1-C), these results together suggest that (CKPV)_2_ could effectively prevent above acute inflammations.

## Discussion and Conclusions

Our results showed that (CKPV)_2_ dose-dependently inhibited Candida albicans colonies formation. In a rat Candida albicans vaginitis model, (CKPV)_2_ administration significantly inhibited vaginal Candida albicans survival and induced macrophage M_2_ polarization. (CKPV)_2_ inhibited Candida albicans phagocytosis by primary cultured macrophages. Further, (CKPV)_2_ promoted macrophages cAMP production through activating MC1R. In macrophages, (CKPV)_2_ administration inhibited the production of pro-inflammatory cytokines including TNF-α, IL-1β and IL-6, while increasing arginase activity and the secretion of anti-inflammatory cytokines (IL-10), favoring a M1 to M2 polarization. These effects by (CKPV)_2_ on macrophages were almost reversed by MC1R siRNA knockdown. Our evidence suggest that the synthetic melanocortin (CKPV)_2_ exerts both anti-fungal and anti-inflammatory activities against Candida albicans vaginitis, probably through regulating macrophages M1 to M2 polarization.

Local immunity plays a decisive role in the vaginal mucous Candida infections. Studies confirmed that the mice vaginal mucosa contained a large number of epithelial cells, dendritic cells, langerhans cells, neutrophils, macrophages and T cells [41]. Neutrophils are recognized as the dominant ones in innate immune cells of vagina. However, studies have indicated that neutrophils were not main ones to clear the yeast burden during the infection, although they have shown abilities of yeast phagocytosis [42]. Other innate immune cells such as monocytes and NK cells were also found in infected vaginal cavity [42]. Mononuclear cells can further differentiate into macrophages which play an important role in innate and adaptive immunity against microbial of host defense [40]. Monocytes/macrophages are able to “eat” extraneous pathogen, eliminate the aging and injured cells, destroy tumor cells and participate in immune responses [40]. Macrophages have the enormous phagocytic capacities, and can be divided into two subtypes namely M1 and M2 [43]. M1-polarized macrophages could activate an inflammatory response via secreting granzyme, porforin and pro-inflammatory cytokines including IL-1β, IL-6 and TNF-α [44]. Therefore, they are beneficial during the early stage of infection by eliminating pathogens, but may cause adverse effects through activating sustained inflammation response latter. M2-polarized macrophages secrete IL-10 and have high arginase activity to promote tissue restoration and reconstruction [40], [45]. As such, polarization of macrophages is critical in the inflammatory reactivation outcomes [40], [45]. Our immunohistochemistry results showed that the infiltrated inflammatory cells of Candida albicans vaginitis model rats were mainly M1 subtype macrophages (CD68 positive), after (CKPV)_2_ administration, these macrophages were mainly M2 positive (CD163 positive). Our results also showed that (CKPV)_2_ significantly inhibited macrophage phagocytosis and the production of pro-inflammatory cytokines(IL-1β,IL-6 and TNF-α). Meanwhile, (CKPV)_2_ enhanced IL-10 secretion and arginase activity in primary cultured macrophages, indicating a typical macrophageM1-to-M2 polarization.

Melanocortin receptor 1 (MC1R), a G protein coupled receptor, can be activated by melanocortin peptides [46]. It widely participates in inflammatory and immune responses through activating downstream signals including adenylate cyclase (AC) [32], [47], [48]. Previous studies have confirmed that anti-inflammatory effects of α-MSH were mainly depend on the MC1R [49], [50]. Our results here suggested that (CKPV)_2_-induced cAMP production and anti-inflammatory and anti-fungal abilities were also dependent on MC1R, as MC1R RNAi knockdown almost blocked those effects.

### Conclusions

(CKPV)_2_ showed an excellent anti-fungal and anti-inflammatory effect against Candida albicans vaginitis both in vivo and in vitro. (CKPV)_2_ inhibits macrophage infiltration in vaginal mucosa tissues and induces the macrophages M1 to M2 polarization.

## Supporting Information

Figure S1 **Anti-acute inflammatory effects of (CKPV)_2._** (A) (CKPV)_2_ inhibits mouse ear edema induced by xylene. The inhibitory rate was determined from the discrimination between the two ears of each mouse. (B) (CKPV)_2_ inhibits rat paw edema induced by albumen. Volumes of paws were measured at 0.5, 1, 2, 4 and 6 h after 0.05 ml albumen (10%) treatment. (C) (CKPV)2 inhibits rat foot itching induced by phosphate. The total amount of given histamine phosphate was the itching limens. Experiments in this figure were repeated three times and similar results were obtained. There was a significant difference between model and drug treatment groups. *p<0.01 (ANOVA).
(EPS)

Text S1 **Protocol of acute inflammation models used in this study.**
(DOC)
