# Supplementary material for: The Synthetic Melanocortin (CKPV)2 Exerts Anti-Fungal and Anti-Inflammatory Effects against Candida albicans Vaginitis via Inducing Macrophage M2 Polarization
Source: PLoS One. 2013 Feb 14;8(2):e56004. doi: 10.1371/journal.pone.0056004 (PMC3573073; doi:10.1371/journal.pone.0056004)
Supplement: Text S1 — Protocol of acute inflammation models used in this study. (DOC) [file pone.0056004.s002.doc]

**Text S1:**

**Protocol of acute inflammation models used in this study**

**Mouse ear edema**

Sixty ICR female mice were divided into 6 random groups, the right ears were administrated with blank matrix gel, dexamethasone gel (Sanjiu Pharmaceutical Co. Ltd , Shenzhen, China) (3.5mg/kg), α-MSH gel (5 mg/kg), (CKPV)_2_ gel (15 mg/kg, 7.5 mg/kg, 2.5 mg/kg) per day for two days. At day 3, 30 min after the drug administration, the right ear of each mouse was washed and incubated with 20 μl xylene (Ling Feng Chemical Reagent Company, Shanghai, China). Afterwards, the mice were sacrificed, both ears (control and treatment) of each rat were cut off and weighed immediately . The inhibitory rate of each drug was determined by calculating the discrimination between the two ears of each mouse.

**Rats paw edema**

Forty-eight female rats were divided into 6 random groups, the right paws were administrated with blank matrix, dexamethasone gel (2.5 mg/kg), α-MSH gel (3.5 mg/kg) or (CKPV)_2_ gel (10 mg/kg, 5 mg/kg and 2 mg/kg ) per day for two days. At day 3, the right paws were subject to 0.05ml albumen (10%) treatments, volumes of paws were measured at 0.5, 1, 2, 4 and 6h respectively .

**Rat foot itching**

Forty-eight SD female rats were divided into 6 random groups, and each right paw was treated daily with blank matrix, dexamethasone gel (2.5 mg/kg), α-MSH gel (3.5 mg/kg), (CKPV)_2_ gel (10 mg/kg, 5 mg/kg and 2 mg/kg ) for two days. At day 3, the right paws were administrated with 0.05 ml various concentrations of histamine phosphate (0.01 %-1.0 % Aladdin, Shanghai, China) every 3 min until the rats turned back and licked the right paws. The total amount of given histamine phosphate at this time was the itching limens .

Reference

1. Macaluso A, McCoy D, Ceriani G, Watanabe T, Biltz J, et al. (1994) Antiinflammatory influences of alpha-MSH molecules: central neurogenic and peripheral actions. J Neurosci 14: 2377-2382.

2. Gordon JS, Wolanin PM, Gonzalez AV, Fela DA, Sarngadharan G, et al. (2008) Topical N-acetyl-S-farnesyl-L-cysteine inhibits mouse skin inflammation, and unlike dexamethasone, its effects are restricted to the application site. J Invest Dermatol 128: 643-654.

3. Wang T, Fu F, Zhang L, Han B, Zhu M, et al. (2009) Effects of escin on acute inflammation and the immune system in mice. Pharmacol Rep 61: 697-704.

4. Wise LE, Cannavacciulo R, Cravatt BF, Martin BF, Lichtman AH (2008) Evaluation of fatty acid amides in the carrageenan-induced paw edema model. Neuropharmacology 54: 181-188.

5. Simons KJ, Watson WT, Chen XY, Simons FE (1989) Pharmacokinetic and pharmacodynamic studies of the H1-receptor antagonist hydroxyzine in the elderly. Clin Pharmacol Ther 45: 9-14.

6. Bernstein JE, Whitney DH, Soltani K (1981) Inhibition of histamine-induced pruritus by topical tricyclic antidepressants. J Am Acad Dermatol 5: 582-585.
